# The Physicians’ Hand-Book for 1891

**Published:** 1891-02

**Authors:** 


THE PHYSICIANS' HAND-BOOK FOR 1891. By ALBERT D. ELMER, M. D. New York : G. P. Putnam's Sons. *The Knickerbocker Press*, 1891.

This book has been a favorite with the profession for over thirty years, combining the features of a visiting list, account book and hand-book of practice. The W. A. Townsend Publishing Co. has transferred its publication to the Putnam's, and that means no falling off in the mechanical execution and all that pertains to good work on the part of the publishers.

The hand-book contains some unique features and ought to be examined by every physician.

